# In Vitro Study of a Novel Nanogold-Collagen Composite to Enhance the Mesenchymal Stem Cell Behavior for Vascular Regeneration

**DOI:** 10.1371/journal.pone.0104019

**Published:** 2014-08-05

**Authors:** Huey-Shan Hung, Chih-Hsuan Chang, Chen-Jung Chang, Cheng-Ming Tang, Wei-Chien Kao, Shinn-Zong Lin, Hsien-Hsu Hsieh, Mei-Yun Chu, Wei-Shen Sun, Shan-hui Hsu

**Affiliations:** 1 Graduate Institute of Basic Medical Science, China Medical University, Taichung, Taiwan. R.O.C.; 2 Center for Neuropsychiatry, China Medical University Hospital, Taichung, Taiwan, R.O.C.; 3 Central Taiwan University of Science and Technology, Department of Medical Imaging and Radiological Science, Taichung, Taiwan, R.O.C.; 4 Institute of Oral Sciences, Chung Shan Medical University, Taichung, Taiwan, R.O.C.; 5 China Medical University Beigang Hospital, Yunlin, Taiwan, R.O.C.; 6 Graduate Institute of Immunology, China Medical University, Taichung, Taiwan, R.O.C; 7 Blood Bank, Taichung Veterans General Hospital, Taichung, Taiwan, R.O.C.; 8 Institute of Polymer Science and Engineering, National Taiwan University, Taipei, Taiwan, R.O.C.; 9 Rehabilitation Engineering Research Center, National Taiwan University, Taipei, Taiwan, R.O.C.; Université de Technologie de Compiègne, France

## Abstract

Novel nanocomposites based on type I collagen (Col) containing a small amount (17.4, 43.5, and 174 ppm) of gold nanoparticles (AuNPs, approximately 5 nm) were prepared in this study. The pure Col and Col-AuNP composites (Col-Au) were characterized by the UV-Vis spectroscopy (UV-Vis), surface-enhanced raman spectroscopy (SERS) and atomic force microscopy (AFM). The interaction between Col and AuNPs was confirmed by infrared (IR) spectra. The effect of AuNPs on the biocompatibility of Col, evaluated by the proliferation and reactive oxygen species (ROS) production of mesenchymal stem cells (MSCs) as well as the activation of monocytes and platelets, was investigated. Results showed that Col-Au had better biocompatibility than Col. Upon stimulation by vascular endothelial growth factor (VEGF) and stromal derived factor-1α (SDF-1α), MSCs expressed the highest levels of αvβ3 integrin/CXCR4, focal adhesion kinase (FAK), matrix metalloproteinase-2 (MMP-2), and Akt/endothelial nitric oxide synthase (eNOS) proteins when grown on the Col-Au (43.5 ppm) nanocomposite. Taken together, Col-Au nanocomposites may promote the proliferation and migration of MSCs and stimulate the endothelial cell differentiation. These results suggest that Col-Au may be used to construct tissue engineering scaffolds for vascular regeneration.

## Introduction

Vascular grafts are widely used for a number of medical treatments. The mechanical and biological properties are of crucial concerns for vascular graft materials [1]. These properties are difficult to achieve with the current vascular grafts that are made of synthetic polymers such as expanded polytetrafluoroethylene (ePTFE) and polyethylene terephthalate (PET) [2]. Although these inert, stiff polymeric grafts successfully replace large arteries, problems arise with small-diameter vessels resulting in high stenosis rates [3]. Major risk factors leading to stenosis include unsatisfactory biocompatibility [4] and lack of endothelialization capacity [5]. In the more recent tissue engineering approach, cells are induced to produce collagen, elastin, or other matrix components. This approach has been considered promising as to overcoming the limitation of the current vascular grafts [6].

Investigators have attempted to create a confluent monolayer of endothelial cells (ECs) on the luminal surface of the vascular grafts in order to mimic the natural blood vessels [7]–[9]. However, the source of ECs is rather limited. Mesenchymal stem cells (MSCs) was shown to home to various sites of injury when injected into the vascular system. Besides, they reside within the connective tissue of most organs [10]. Recent reports proposed that MSCs may function as vascular pericytes and identify the vascular tube as their stem cell niche [11]. Many encouraging publications showed the differentiation of MSCs across the wounded tissue. MSCs may promote tissue repair by secreting factors that enhance regeneration of injured tissues, stimulate proliferation and differentiation of endogenous stem cell-like progenitors, and decrease inflammatory and immune reactions [12], [13].

Considerable efforts have been made to develop suitable scaffolds for tissue engineering application using biodegradable polymers, collagen, and polymer/collagen blends. Since collagen is a basic structural element in native extracellular matrices, its presence is abundant in natural tissues, composing 30% by weight of body protein tissues [14]. It makes a natural choice for biomedical materials and tissue-engineering matrices [15]. Collagen has a high density of the RGD (Arg-Gly-Asp) sequence and other functional sequences for cell adhesion and differentiation [16]–[18]. The possibility to become a three-dimensional matrix makes collagen attractive in many therapeutic applications [19]–[23]. The extracted type I collagen, under appropriate conditions in vitro, can spontaneously self-assemble to form biodegradable and biocompatible insoluble fibrils of high mechanical strength and low immunogenicity [24]–[26]. However, collagen matrix lacks the mechanical properties desired for vascular grafts [27], [28]. When used as vessel substitutes in vivo, they need to be modified for high compliance, elongation, and strength. In addition, collagen has poor antithrombotic activity [29].

The adhesion and proliferation of ECs and MSCs on vascular grafts are generally poor. Gold (Au) is highly biocompatible [30].It has been used for crosslinking biomolecules to produce a biomimetic interface to control cellular behavior [31], [32]. In our previous studies, the nanocomposite of synthetic polyurethane (PU) incorporated with nanogold (AuNPs) demonstrated good biocompatibility [33]–[36]. To improve the properties of collagen for vascular applications, AuNPs were incorporated into natural collagen matrix to create a biomimicking environment for the native growth of MSCs for vascular graft applications.

## Materials and Methods

### 2.1. Preparation of collagen-nanocomposites (Col-Au)

Type I Collagen (Col) was purchased from BD Bioscience (USA). Col-Au nanocomposites were prepared by mixing 0.5 mg/ml solution of Col with a fixed amount of AuNPs (Gold Nanotech Inc, Taiwan). AuNPs were obtained from bulk gold which was atomically vaporized by an electrically gasified method under vacuum condition. AuNPs were accumulated in a cold trap system and collected by centrifugation [37]. The size of the AuNPs could be controlled by the evaporation time and electric current. This method rendered negatively charged (bare) AuNPs in distilled water without using any surfactant or stabilizer. The diameter of the AuNPs used in this study was ∼5 nm. Col-Au nanocomposites (denoted “Col-Au”) contained 17.4, 43.5, or 174 ppm AuNPs in the final dry weight. The solution was cast to culture dish or 15 mm round coverslip glass (20 µg/cm^2^). Collagen (Col) was dissolved at 1 mg/ml in 5 mM acetic acid at 4°C overnight. The solution was diluted 1∶1 with 2×PBS, resulting in a 0.5 mg/ml solution pH 7.3, and placed in a water bath at 21°C. The temperature of the bath was allowed to increase to 37°C at which point the solution was returned to 4°C for storage. AuNPs were then added to Col solution (0.5 mg/ml) so the final Col-Au contained 17.4, 43.5 and 174 ppm of AuNPs. Col and Col-Au nanocomposites were coated to the cell culture tissue dish, cell culture plate, or 15 mm round coverslip glass at the amount of 20 µg/cm^2^ to form Col and Col-Au thin films.

### 2.2. Characterization of Col and the Col-Au nanocomposites

The UV/Vis spectra of Col and Col-Au were analyzed by a spectrophotometer (Helios Zeta, Thermo Fisher, USA). The infrared (IR) spectra was obtained by a Fourier transform IR spectrometer (Shimadzu Pretige-21, Japan). Each sample was scanned eight times in the spectral area of 400–4000 cm^−1^ with a resolution setting of 2 cm^−1^ and averaged to produce each spectrum. Col solution containing different amounts of AuNPs (17.4, 43.5 and 174 ppm) in 0.5 ml of PBS was incubated in a 37°C water bath for 1 h. The samples were then deposited on clean glass-bottom petri dishes. All surface-enhanced Raman spectroscopy (SERS) experiments were performed in liquid and processed by 3D Nanometer Scale Raman PL Microspectrometer (Nanofinder®30)(Tokyo Instruments, Japan). The surface of dried materials was analyzed by an atomic force microscope (AFM) equipped with a 100-µm piezoelectric scanner (JEOL JSM-6700 F, Japan). The images were processed in the tapping mode in air with a triangular cantilever. Topography images were recorded and the average roughness was calculated based on the images.

### 2.3. Adhesion and cytoskeleton of MSCs

To observe the cytoskeleton and cell morphology, MSCs were seeded at a density of 0.5×10^4^ cells per well after 48 h of incubation in 24-well plates containing Col and Col-Au on coverslip glass. Cell cultured on coverslip glass was used as control. For cytoskeletal staining, cells were fixed with 4% paraformaldehyde (PFA)/with phosphate buffered saline (PBS) for 15 min and permeabilized with 0.5% (v/v) Triton-X 100 (Sigma, USA) in PBS for 10 min prior to staining. After then, 5% (v/v) of bovine serum albumin (BSA) (Sigma, USA) was used to block the non-specific binding. Finally, cells were stained with optimal concentration of rhodamine phalloidin (∼6 µM) (Sigma, USA) for 30 min. The cell nuclei were then stained with 4, 6-diamidino-2-phenylindole (DAPI) (1 µg/ml) for 10 min. Cell morphology was observed by a scanning electron microscope (SEM) (JEOL JSM-6700 F, Japan) after fixation, dehydration, critical-pointed dried, and sputter-coated with gold.

### 2.4. Proliferation and ROS generation of MSCs

MSCs were collected from human umbilical cord Wharton’s jelly tissue were kindly provided by Prof. Woei-Cherng Shyu [38] and maintained in condition medium [high glucose Dulbecco’s Modified Eagle’s Medium (DMEM) (Invitrogen), supplemented with 1% (v/v) antibiotics 100 U/ml penicillin/streptomycin, 1% sodium pyruvate]. Cells of passages 8–20 maintained the correct phenotype [36] and were used in this study. Two hundred µl of MSCs suspension with a density of 6×10^3^ cells/ml was seeded into each well of the culture plates after Col and Col-Au were coated into the bottom of 96-well tissue culture plates. Cells cultured tissue culture polystyrene (TCPS) was used as a control group. After 24, 48, and 72 h of incubation, the adherent cells were analyzed by the MTT assay. Briefly, [3-(4, 5-cimethylthiazol-2-yl)-2, 5-diphenyl tetrazolium bromide] (MTT) (0.5 mg/ml) solution was applied in each well of the culture plate and incubated at least for 3–4 h at 37°C incubator. After then, dimethyl sulfoxide (DMSO) was added to dissolve the crystals and the absorbance at 570 nm was read by a microplate reader (SpectraMax M2, Molecular Devices, USA).

The generation of intracellular reactive oxygen species (ROS) was measured using the oxidation-sensitive fluorescent dye of 2, 7-dichlorofluorescin diacetate (DCF-dA) (Sigma). MSCs (2×10^5^/well) were seeded in 6-well of Col or Col-Au coated culture plates for 48 hr. After incubation, cells were washed twice with PBS buffer and incubated with 500 µl of PBS containing 20 µM of DCF-dA at 37°C incubator for 60 min. After then, cells were measured at 530 nm emission wavelength after excitation at 480 nm at 30-min intervals for up to 4 h using the FACS Calibur flow cytometer (Becton Dickinson, USA). The generation of intracellular reactive oxygen species (ROS) as indicted an increase in fluorescence intensity. Fluorescein-positive cells were evaluated using the FCS software (Becton Dickinson, USA).

### 2.5. Monocyte and platelet activation tests

The protocols were approved by the Institutional Review Board (IRB, approval number CE12164; Taichung Veterans General Hospital Taichung) with written informed consent obtained from the blood donors. The standard procedure of monocyte isolation from human blood sample of volunteer donors and culture method were described previously [34], [39]. Cells were suspended in a medium of RPMI containing 10% FBS and 1% (v/v) antibiotic (10,000 U/ml penicillin G and 10 mg/ml streptomycin) and the cell concentration was adjusted to 1×10^5^ cells/ml. One ml cell suspension was added to each well and allowed to adhere for 96 h at 37°C in 5% CO_2_. After then, the adherent cells were trypsinized and the numbers of monocytes and macrophages were counted based on their morphology by an inverted phase contrast microscope. The ratio between numbers of monocytes and macrophages (i.e. the conversion ratio) was used as an inflammatory index. Additionally, the phenotypic marker of macrophage, CD 68, was stained by immmunofluorescence by the anti-CD68 antibody (GeneTex InC, USA).

Platelet-rich plasma (∼2×10^6^ platelets/ml) was added in a 24-well culture plate containing Col and Col-Au. The plasma was removed after incubation for 1 h and the number of adherent platelets were counted by a cell counter (Metasizer, Coulter, USA). To examine the platelet morphology, samples were subjected to the standard procedures before SEM observation. Each platelet was identified for the morphological change and the degree of activation was calculated by the standard formulation: 0 = round (in unactivated type), 1/4 = dendritic (in pseudopodial but no flattening type), 1/2 = spread-dendritic (in flattened pseudopodia type), 3/4 = spreading (in late phase pseudopodial with hyaloplasm spreading type), and 1 = fully spread (in totally activated type). The average platelet activation was quantified based on 50 adherent platelets.

### 2.6. Protein expressions of MSCs on Col and Col-Au

Cells were grown in basal medium as previously described or in the medium containing recombinant human vascular endothelial growth factor (VEGF) (ProSpec-Tany TechnoGene Ltd, USA) (50 ng/ml) or recombinant human stromal derived factor-1 alpha (SDF-1α) (ProSpec-Tany TechnoGene Ltd, USA) (50 ng/ml). The expression of αvβ3 integrin and eNOS expression was visualized by immunofluorescence staining. MSCs (2×10^4^ cells in each well) were incubated for 48 h. Cells were fixed, permealized, and further washed with PBS. The primary antibodies [primary anti-αvβ3 integrin antibody (Santa Cruz), primary anti-eNOS integrin antibody (Santa Cruz) and primary anti-CD31 antibody (Santa Cruz)] were added to the culture plate overnight. Secondary antibodies [FITC-conjugated antibody (green color fluorescence) or Cy5.5-conjugated immunoglobulin (red color fluorescence)] were added to the sample for 60 min. The cell nuclei were stained with DAPI (1 µg/ml) for 10 min.

Western blots were conducted to measure the expression of p-FAK, p-Akt, total FAK, and total Akt. After 48 h of incubation, cells were washed three times with the ice-cold PBS, the adherent cells were collected by trypsin (0.025%) treatment. After centrifugation, cell pellet were suspend in the RIPA lysis buffer, rotated for 1 h at 4°C, and centrifuged for 15 min at 14000×g. The protein supernatant were collected and concentration was quantified by using a protein assay kit (Bio-Rad Labs, USA). Total amount of protein sample (30 µg) of each sample were processed to sodium dodecyl sulfate polyacrylamide gel electrophoresis (SDS-PAGE) and separated proteins were then transferred onto a nitrocellulose membrane by a semi-dry blotting technique. After then, membrane was blocked with 5% non-fat dry milk in PBS for 1 h at room temperature before overnight incubation at 4°C with the primary anti-phospho-FAK (Tyr-576/577) antibody (1∶500 dilution, Cell Signaling), anti-phospho-Akt (Ser-473) antibody (1∶500 dilution, Cell Signaling) and loading controls [anti-total-FAK antibody (1∶2000 dilution, Cell signaling); anti-total-Akt antibody (1:2000 dilution, Cell Signaling] to ensure standard of loading control. The membranes incubated with peroxidase-conjugated secondary antibodies were reacted with the ECL western blotting detection system (Amersham Life Science, USA). Qquantification was performed by densitiometric analysis with the LabWork Image Acquisition and Analysis software.

Gelatin Zymogrpahy was performed to identify the expression of matrix metalloproteinases (MMPs). Cell culture condition media at 48 h were collected and subjected onto the gels without thermal denaturation. After then, samples (about 10–25 µl) were loaded and electrophoresis was performed with 1X Tris-Glycine SDS buffer according to the standard running conditions (∼125 V, constant voltage, 120 min). After electrophoresis, running gels were removed and incubated for 1 h at room temperature and in the 100 ml of Zymogram renaturing buffer on a shaker to remove SDS. Gelatin impregnated gels were rinsed with water and stained with 100 ml with 0.5% Coomassie brilliant blue R-250 buffer and destained with 10% acetic acid buffer. The protease activity expression area will show as bright bands against a dark blue background where the protease has digested the substrate. The expression intensity of MMP gelatinase activity was then calculated by quantitative densitometry. Data were normalized on the protein amount measured in cell culture conditio medium. Images were analyzed by Image Pro Plus 5.0 software (Media Cybernetics).

### 2.7. Migration ability of MSCs

The cell migration assay was performed using Oris Cell Migration Assay reagent kit (Platypus Technologies, Madison WI, USA). Col or Col-Au coated coverslip glass were placed into each well of an Oris Cell Migration Assay Tri-Coated plate. Cell seeding stoppers (2 mm in diameter) were inserted in each well on top of various materials coating to prevent cell adhesion in the center region of the well. Cell seeding stopper was then placed in the central area on top of various coating. Oris seeding stoppers were seeded with 100 µl/well (8×10^3^ cells/ml) and incubated for 48 h incubation to reach confluent. The stopper was then removed from the test well, but remained in place in the pre-migration reference wells. The seeded plate was incubated in 37°C for observation of pre-migration (t = 0 h) and post-migration (24 to 48 h). Cell population in the end-point assays were stained with Calcein AM (Sigma) (200 µl and 2 µM) containing serum free medium and the image was captured by a fluorescence microscope (Zeiss Axio Imager A1, USA). Cells that migrated in the detection zones were quantified by measuring fluorescence intensity and analyzed by Image Pro Plus 5.0 software.

### 2.8. Statistical analysis

Multiple samples (n = 3∼6) were collected in each experiment and results were expressed as mean ± standard deviation. All experiments were repeated independently for at least three time to assure for reproducibility. Single-factor analysis of variance (ANOVA) method was used to assess the statistical significance of the results. *p* values less than 0.05 are considered significant.

## Results

### 3.1. Characterization of Col and Col-Au nanocomposites

**Figure 1:**
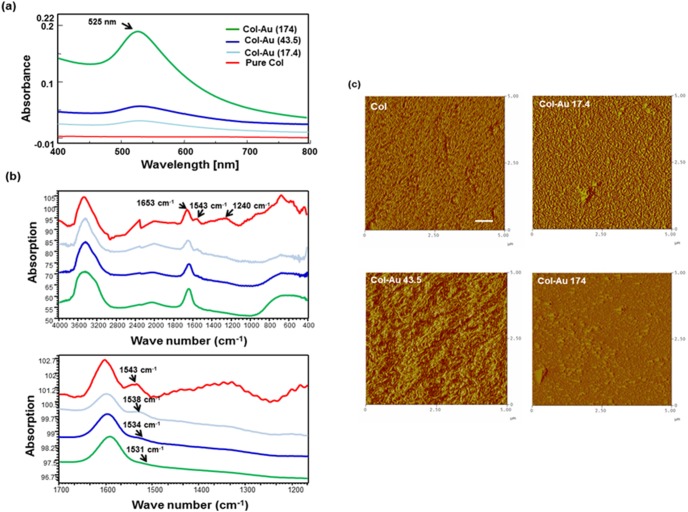
Materials Characterization. (**a**) UV–Vis absorption spectra for Col solution ater loading of different concentrations of AuNPs (∼17.4 ppm, 43.5 ppm, and 174 ppm). (**b**) IR spectra of Col and Col-Au nanocomposites in the total wavenumber ranges from 400 cm^−1^ to 4000 cm^−1^ region and from 1200 cm^−1^ to 1700 cm^−1^. All results are representative of one of three independent experiments. (**c**) AFM topography diagrams for the pure Col, and Col-Au nanocomposites containing 17.4 ppm, 43.5 ppm, and 174 ppm of AuNPs. All results are representative of one of three independent experiments. Ra is the average roughness of the sample. Scale bar = 100 nm.

The UV-Vis absorption peak at 525 nm showed up after loadings different concentration of AuNPs (∼17.4ppm, 43.5 ppm, and 174 ppm) to Col in a dose-dependent manner (**Figure 1a**). The IR spectra (**Figure 1b**) for Col had a peak at 1635 cm^−1^, corresponding to the amide I vibration band. The amide II and amide III adsorption peaks, typical of pure Col, were located at 1543 cm^−1^ and 1240 cm^−1^ [40]. After loading AuNPs, the intensities of these amide bands significantly decreased. The amide II peak position at 1543 cm^−1^ in pure Col was shifted to 1538 cm^−1^ in Col-Au 17.4 ppm, to 1534 cm^−1^ in Col-Au 43.5 ppm, and to 1531 cm^−1^ in Col-Au 174 ppm. The other amide bands became almost invisible. Surface topography of Col and Col-Au nanocomposites was examined by AFM (**Figure 1c**). In literature, the self-assembled collagen fibers showed a bimodal distribution of diameters approximately at 15 nm and 100 nm characterized by the AFM [41]. In this study, we observed a dense network of fibers in pure Col and Col-Au 17.4 ppm. Moreover, the distinct sheet of fibrils was observed in Col-Au 43.5 ppm. Aggregation, on the other hand, was observed for Col-Au 174 ppm, possibly associated with the higher concentration of AuNPs. The surface of pure Col had an average roughness (Ra) value of 14.53±4.3 nm and was more homogenous. Ra increased in the presence of AuNPs. At 43.5 ppm of AuNPs, Ra was significantly greater (107.8±13.9 nm) than the other materials (p<0.05). Ra was close between Col-Au 17.4 ppm (37±6.7 nm) and the Col-Au 174 ppm (32±5.0 nm). In this study, we observed a dense network of fibers in pure Col and Col-Au 17.4 ppm. Moreover, the distinct sheet of fibrils was observed in Col-Au 43.5 ppm. Aggregation, on the other hand, was observed for Col-Au 174 ppm, possibly associated with the higher concentration of AuNPs. The surface of pure Col had an average roughness (Ra) value of 14.53±4.3 nm and was more homogenous. Ra increased in the presence of AuNPs. At 43.5 ppm of AuNPs, Ra was significantly greater (107.8±13.9 nm) than the other materials (p<0.05). Ra was close between Col-Au 17.4 ppm (37.0±6.7 nm) and the Col-Au 17.4 ppm (32.3±5.0 nm).

**Figure 2:**
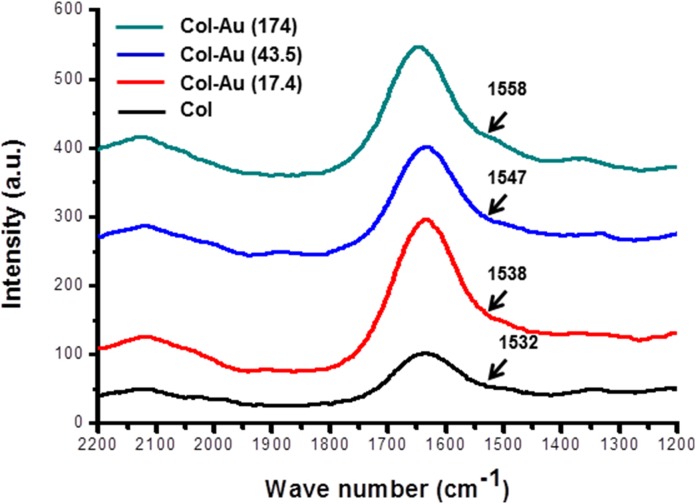
Spectra of Col and Col-Au with peaks in the amide region by surface-enhanced raman spectroscopy (SERS).

The amide I vibration band had a peak position at 1653 cm^−1^ [42], while amide II had peak positions at 1543 cm^−1^ and 1240 cm^−1^ [40]. The Amide II peak position shifted from 1532 cm^−1^ in pure collagen, to 1538 cm^−1^ in Col-Au 17.4 ppm, to 1547 cm^−1^ in Col-Au 43.5 ppm, and to 1558 cm^−1^ in Col-Au 174 ppm (**Figure 2**). The above finding as well as the evidence by FTIR analysis (**Figure 1b**) indicated that the shift of the amide bands intensity was attributed to the incorporation of various amounts of AuNPs. This also implied that adding AuNPs in the collagen matrix may induce more bonding and cause more cellular interaction.

### 3.2. Adhesion and proliferation of MSCs

**Figure 3:**
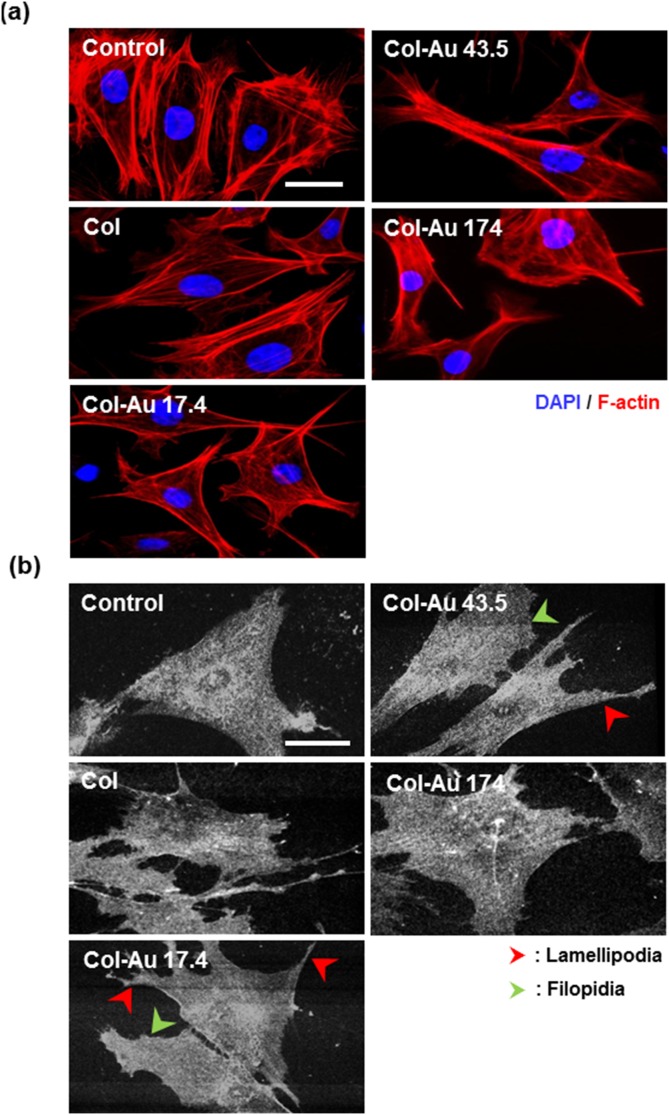
Cytoskeleton and cell morphology. (**a**) Rhodamine phalloidin staining for the cytoskeletal fibers of MSCs on pure Col and different Col-Au nanocomposites at 8 h and 48 h by fluorescence microscopy. Scale bar = 50 µm. (**b**) SEM images for MSCs on pure Col and different Col-Au nanocomposites at 48 h. Arrows indicate filopodia (green color) and lamellipodia (red color). Scale bar = 50 µm.

The cytoskeleton of MSCs on Col-Au examined by phalloidin staining is shown in **Figure 3a**. On control group (glass), Col, and Col-Au 174 ppm, actin fibers exhibited a circumferential shape and were mostly surrounding near the cell body. On Col-Au 43.5 ppm, actin fibers were spread out and more extended with filopodia and lamellipodia. This tendency was also seen on Col-Au 17.4 ppm, but to a lesser extent. The cell morphology was confirmed by SEM images (**Figure 3b**). MSCs on control group (glass), Col, and Col-Au 174 ppm were more circular in shape, while those on Col-Au 43.5 ppm (and Col-Au 17.4 ppm) had significantly more protrusions and were elongated (**Figure 3b**).

**Figure 4:**
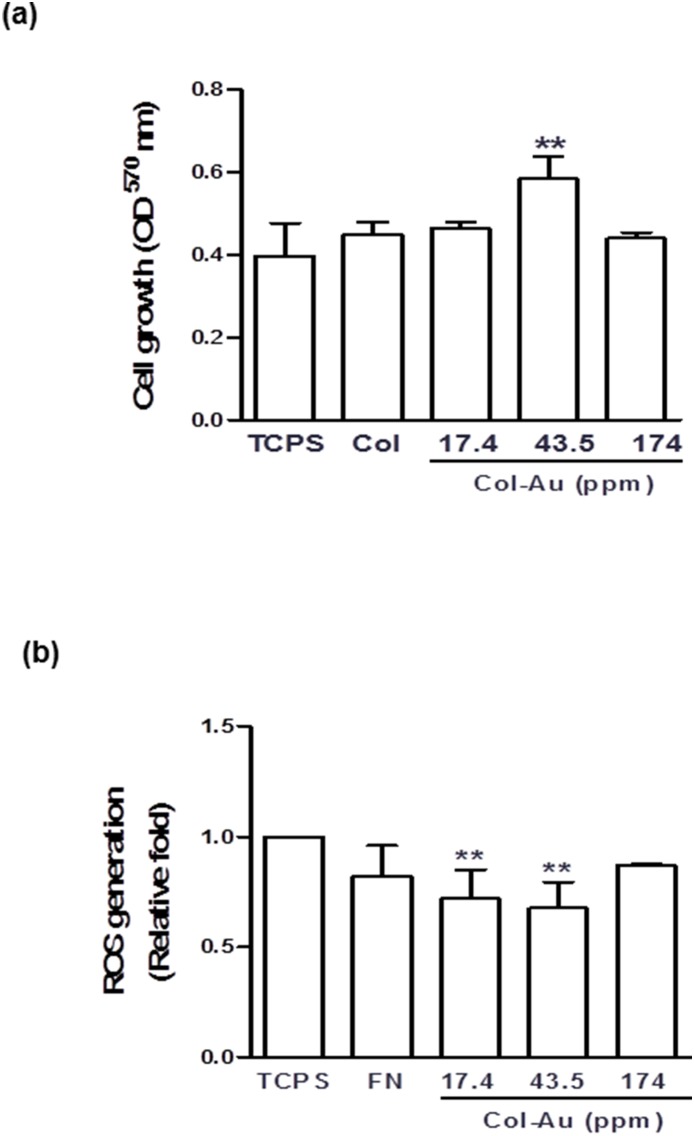
The proliferation of MSCs and reactive oxygen species (ROS) generation assay on different materials after 48 h of incubation. (**a**) MSCs proliferation examined by MTT assay on control (TCPS), pure Col, and Col-Au nanocomposites containing 17.4 ppm, 43.5 ppm, and 174 ppm of AuNPs after 48 h of incubation. **p<0.01: greater than control (TCPS). (**b**) The intracellular ROS quantified by 2, 7-dichlorofluorescein diacetate (DCFH-dA) and flow cytometric analysis. **p<0.01: greater than control (TCPS).

The growth of MSCs on Col and Col-Au after 48 h of incubation are shown in **Figure 4a**. The proliferation ability of MSCs at 48 h was the greatest on Col-Au 43.5 ppm, followed by Col-Au 17.4 ppm, pure Col and Col-Au 174 ppm. The cell number at 24 h or 72 h remained in similar tendencies (**Figure S1)**. ROS generation was lower for Col compared to TCPS and Col-Au 174 ppm (**Figure 4b**), and was the lowest for Col-Au 43.5 ppm, followed by Col-Au 17.4 ppm.

### 3.3. Biocompatibility

**Figure 5:**
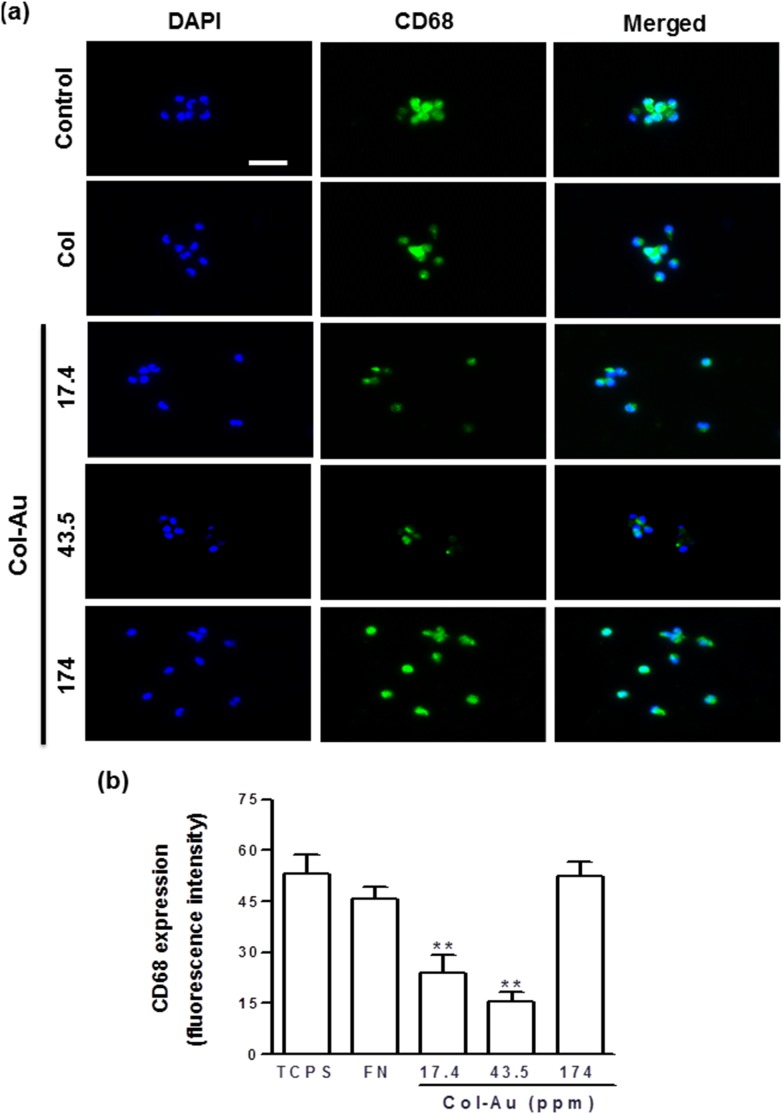
The expression of CD68 for macrophages on different materials at 96 h. (**a**) Cells were immunostained by the primary anti-CD68 antibody and conjugated with FITC-immunoglobulin secondary antibody (green color fluorescence). Cell nuclei were staiend by DAPI (blue color fluorescence). Scale bar = 50 µm. (**b**) CD68 expression was quantified based on fluorescence intensity. **p<0.01: smaller than control (TCPS).

The ratio of human monocytes converted into the activated macrophages by different materials after 96 h is listed in **Table 1**. Col-Au 43.5 ppm had the smallest numbers of monocytes and macrophages and the lowest conversion ratio, followed by Col-Au 17.4 ppm, pure Col, and Col-Au 174 ppm. Semi-quantification was made by CD68 immunofluorescence staining (as a marker of macrophage) (**Figure 5a**). The CD68 fluorescence intensity on Col-Au 43.5 ppm was smaller than those on the other materials (**Figure 5b**). These data indicated that Col-Au 43.5 ppm caused a lower immune response than the other materials.

**Table 1:** Human monocytes adhered and activated on Col and Col-Au nanocomposites.

Materials	Nnumber of monocytes (×10^4^)	Number of macrophages (×10^4^)	Conversion ratio (%)
TCPS (control)	2.36±0.25	0.57±0.31	34.75±0.10
Col	2.39±0.49	0.50±0.10	21.00±0.05*
Col-Au 17.4 ppm	2.05±0.28	0.26±0.03	14.30±0.03**
Col-Au 43.5 ppm	1.93±0.14	0.22±0.05	11.20±0.02**
Col-Au 174 ppm	2.15±0.18	0.51±0.08	23.00±0.02

*p<0.05, greater than TCPS (tissue culture polystyrene);

**p<0.01, greater than TCPS.

**Figure 6:**
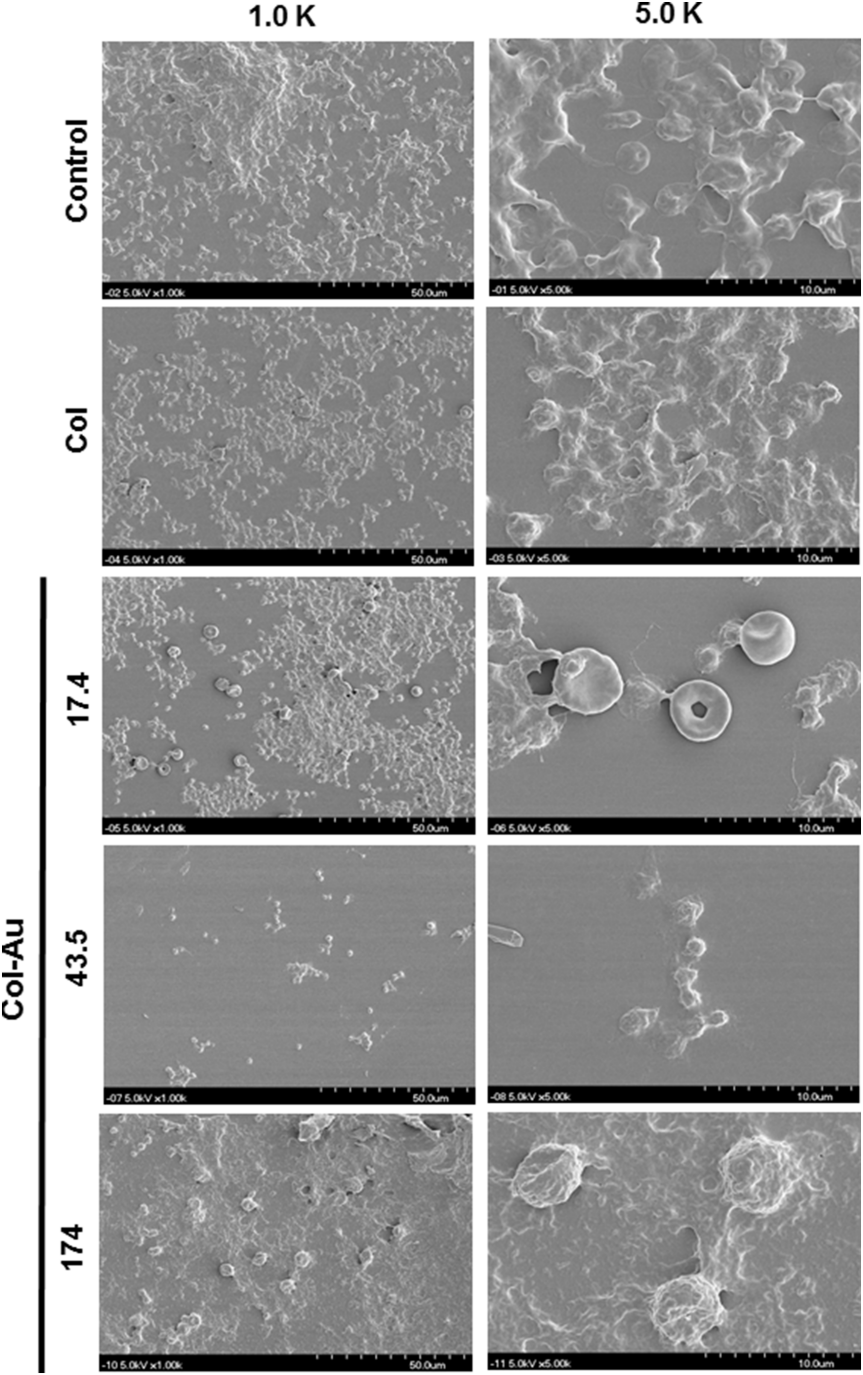
SEM images showing the adhesion and activation of human blood platelets on different matierals.

The platelet activation on Col and Col-Au was examined by SEM images (**Figure 6**). The number of adhered platelets on the control group (glass) was greater than that on pure Col, followed by Col-Au. Moreover, platelets on the control group and on Col were almost flatten (i.e. “activated” platelets). In contrast, platelets on Col-Au 43.5 ppm remained round in shape (“non-activated”). The average scores are listed in **Table 2**. Platelets were less activated on all Col-Au nanocomposites, especially on Col-Au 43.5 ppm (0.12±0.01), followed by Col-Au 17.4 ppm (0.14±0.01), pure Col (0.63±0.10), and Col-Au 174 ppm (0.77±0.03) (p<0.01).

**Table 2:** Scoring of platelet activation on Col and Col-Au nanocomposites.

Materials	Number of adhered Platelets(×10^4^)	Degree of activation(0.0–1.0)
Glass (control)	10.11±2.21	0.91±0.22
Col	9.44±1.00	0.63±0.10
Col-Au17.4 ppm	1.43±0.02**	0.14±0.01**
Col-Au43.5 ppm	1.14±0.06**	0.12±0.01**
Col-Au 174 ppm	7.75±0.27	0.77±0.02

**p<0.01, greater than control (glass).

### 3.4. Differentiation ability of MSCs on Col-Au nanocomposites

**Figure 7:**
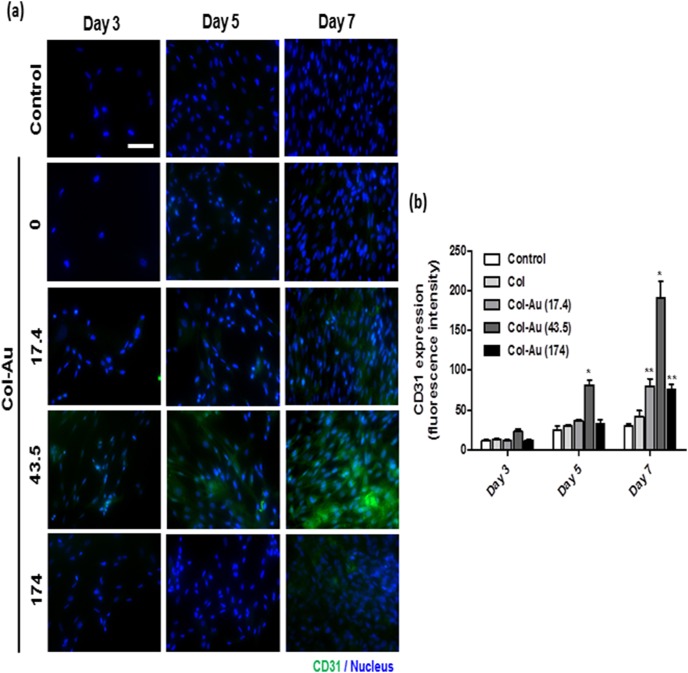
The CD31 protein expression of MSCs on different materials at 3, 5, and 7 days. (**a**) MSCs were immunostained by the primary anti-CD31 antibody and conjugated with FITC-immunoglobulin secondary antibody (green color fluorescence) and cell nuclear staining was performed by DAPI (blue color staining). Results were recorded by fluorescence microscopy. Scale bar = 10 µm. (**b**) Semi-quantitative measurement of fluorescence intensity revealed a significantly higher level of CD31 expression on Col-Au 43.5 ppm. Data are mean ± SD (n = 3). *p<0.01: greater than the other groups [including the control (TCPS), Col, and Col-Au nanocomposites at the other concentrations]; **p<0.01: greater than TCPS and Col.

We further characterized the differentiation ability of MSCs on Col-Au nanocomposites into the EC phenotype. The immunofluorescence expression of CD31 was examined for cells grown on different materials for 3, 5, and 7 days. As evident from the images (**Figure 7a**), the expression of CD31 was clearly observed on Col-Au 43.5 ppm but not was obviously seen on the control group (TCPS) and the pure Col. Semi-quantitative data (**Figure 7b**) showed that, after 5 days and 7 days of incubation, the CD31 expression level on Col-Au 43.5 ppm was significantly higher than any other groups. At 7 days, the expression levels of CD31 on Col-Au 17.4 ppm and Col-Au 174 ppm were also significantly higher than those on TCPS and the pure Col. These results suggested that the Col-Au nanocomposites, in particular Col-Au 43.5 ppm, may induce the differentiation of MSCs into ECs.

### 3.5. VEGF/SDF-1α molecular signaling

**Figure 8:**
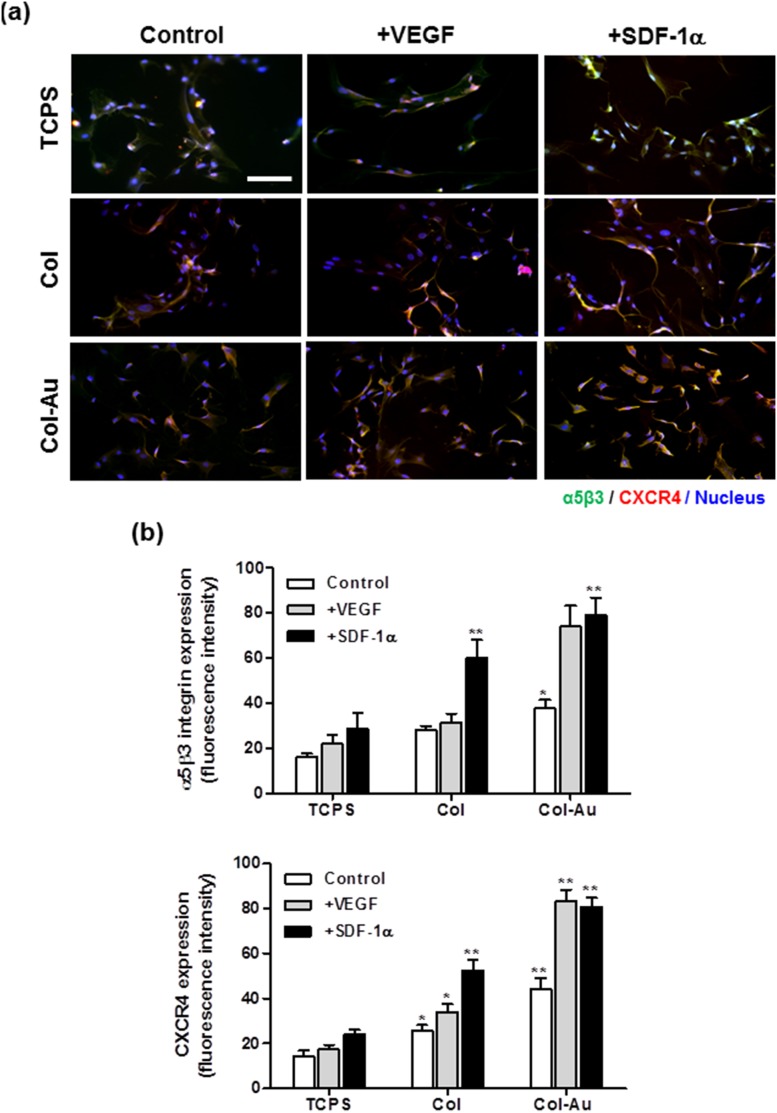
The expression of αVβ3 integrin and CXCR4 for MSCs on different materials at 48 h of incubation and for those treated with either VEGF (50 ng/ml) or SDF-1α (50 ng/ml) in culture media. (**a**) MSCs were immunostained by the primary anti-αvβ3 integrin antibody and primary anti-CXCR4 antibody and conjugated with FITC-immunoglobulin secondary antibody (green color fluorescence), Cy5.5-conjugated immunoglobulin secondary antibody (red color fluorescence). Cell nuclei was stained by DAPI. Scale bar = 100 µm. (**b**) αvβ3 integrin and CXCR4 expressions were quantified based on fluorescence intensity. *p<0.05, **p<0.01: greater than control (TCPS).

Col-Au 43.5 ppm, compared to TCPS and Col, enhanced the αvβ3 integrin (green color) and CXCR4 (red color) fluorescence intensities of MSCs after SDF-1α and VEGF stimulation for 48 h (**Figure 8a**). The expressions of αvβ3 integrin and CXCR4 were co-localized in the cytoplasma. Furthermore, the effect of SDF-1α treatment on their expressions was more prominent, especially on Col-Au 43.5 ppm (**Figure 8b**).

**Figure 9:**
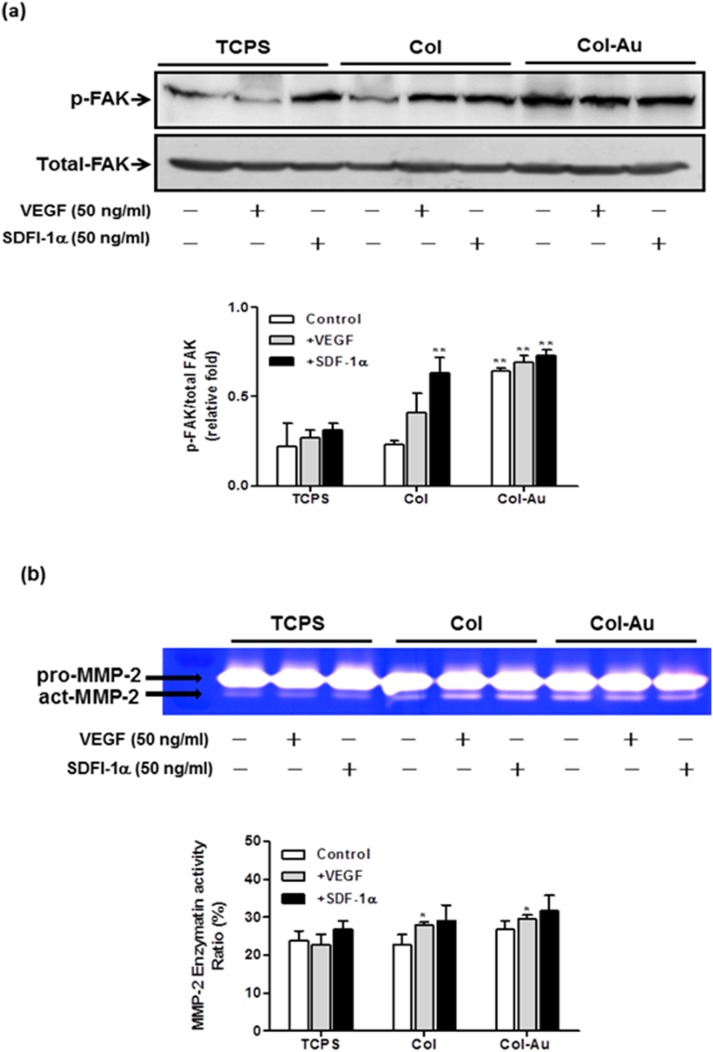
The expression of p-FAK protein and MMP-2 enzymatic activity in MSCs cultured on different materials for 48 h. (**a**) Relative expression ratios of p-FAK was normalized to total FAK. **p<0.01. (**b**) The MMP-2 enzymatic activities was normalized to the protein content. Semi-quantitative measurement of the optical density (OD) of gelatinolytic bands revealed significantly greater MMP-2 expression for MSCs on Col-Au 43.5 ppm. *p<0.05: greater than control (TCPS).

The induction of p-FAK expression was more prominent on Col-Au 43.5 ppm (**Figure 9a**). The treatment with SDF-1α or VEGF further increased the expression. Gelatin zymography showed that the activity of MMP-2 on Col-Au 43.5 ppm was the highest and was further increased after treatment of SDF-1α or VEGF (**Figure 9b**).

**Figure 10:**
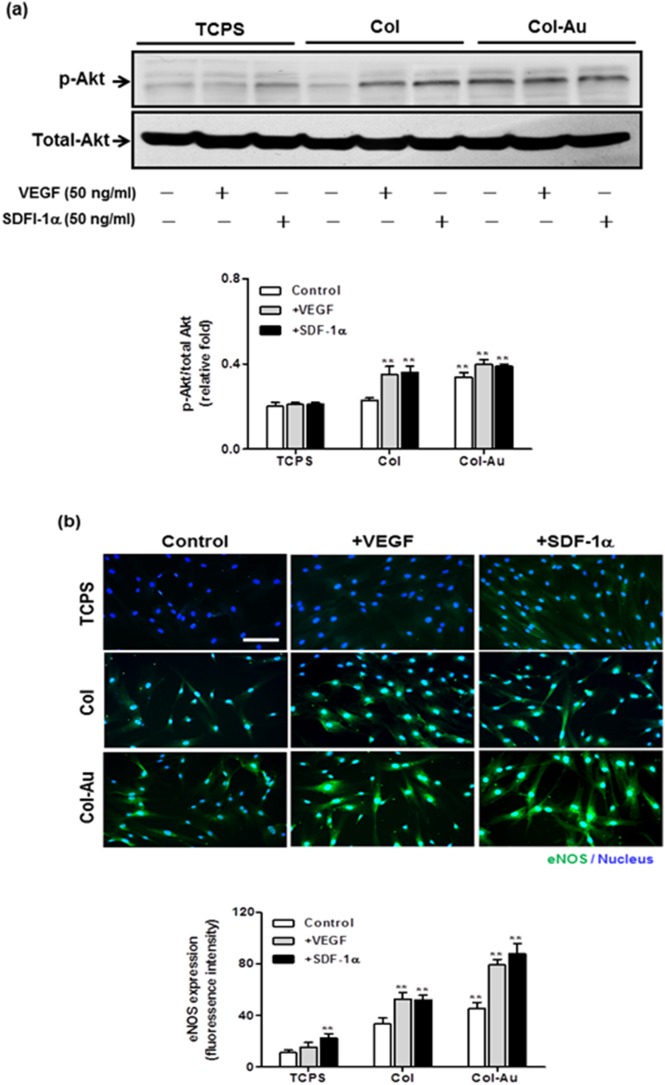
The expression of p-Akt and eNOS proteins in MSCs cultured on different materials for 48 h by western blots and immunofluorescence staining. (**a**) Relative expression ratios of p-Akt was normalized to total Akt. **p<0.01. (**b**) The expression of eNOS protein for MSCs was examined by fluorescence microscopy. Cells were stained with primary anti-eNOS antibody followed by FITC-conjugated immunoglobulin (green color fluorescence). Cell nuclei was stained by DAPI. Scale bar = 10 µm. Semi-quantitative measurement of fluorescence intensity revealed a significantly higher level of eNOS expression compared with the control. *p<0.01: greater than control (TCPS).

The expression of phosphorylated Akt (p-Akt) of MSCs was significantly enhanced on Col-Au 43.5 ppm vs. Col and TCPS (**Figure 10a**). Treatment with SDF-1α or VEGF only increased the expression slightly. The expression and cellular localization of eNOS were shown by the immunofluorescence images (**Figure 10b**). The higher fluorescence intensity of eNOS protein (i.e. EC phenotype) in the cytoplasma was found for MSCs on Col-Au 43.5 ppm, compared with the others. The eNOS expression on Col-Au 43.5 ppm was significantly promoted by SDF-1α or VEGF treatment. Similaryly, SDF-1α treatment induced a more prominent effect on eNOS protein expression than VEGF treatment. These results suggested that Col-Au 43.5 ppm may promote the differentiatin of MSCs into ECs, in particular under the induction of SDF-1α or VEGF.

### 3.6. Cell migration

**Figure 11:**
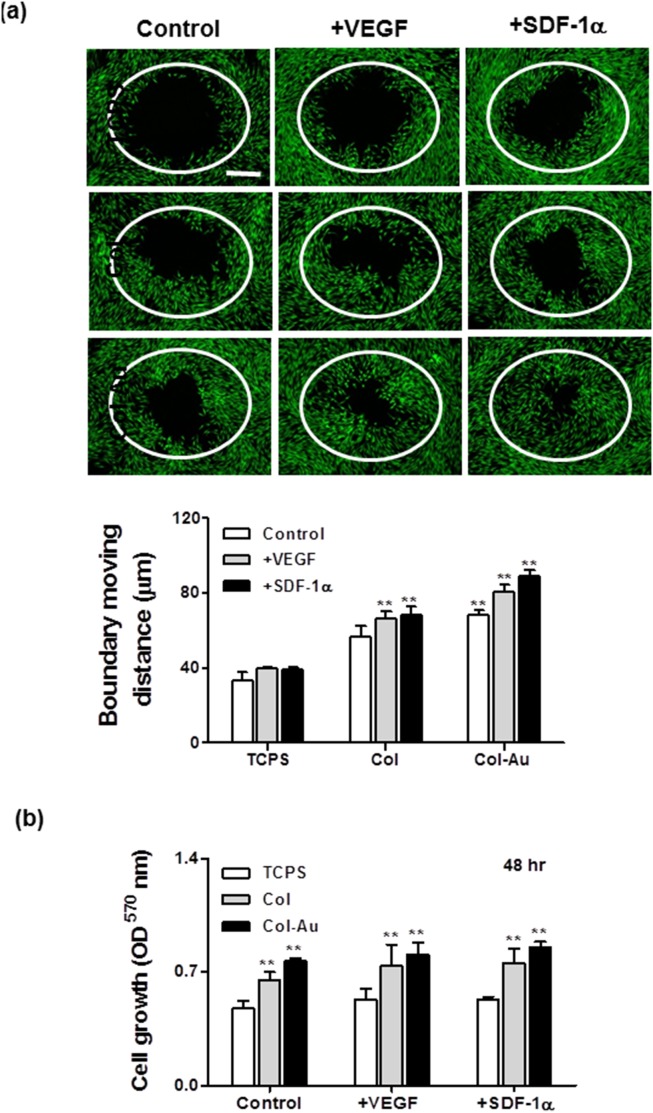
Migration of MSCs on different materials during a period of 48 h. (**a**) Cell migration into the gap zone area was monitored by fluorescence microscopy. Cells were stained by calcein-AM (2 µM) prior to examination. Scale bar = 500 µm. **p<0.01: greater than control (TCPS). (**b**) MSCs proliferation examined by MTT assay. **p<0.01: greater than control (TCPS).

The real-time images of cell boundary captured in pre-migration (t = 0 h) and post-migration (t = 48 h) wells are shown in **Figure 11a**. Since the cells were nearly confluent after 24 h, the boundary moving distance may reflect cell migration ability. It was found that the average moving distance during 0–48 h on Col-Au 43.5 ppm (68.41±2.67 µm) was significantly greater than those on pure Col (56.42±5.95 µm) and control group (TCPS) (33.56±4.55 µm). Moreover, the average moving distance was significantly induced by VEGF (80.46±4.26 µm) or SDF-1α (88.79±3.28 µm) on Col-Au 43.5 ppm. The effects of VEGF and SDF-1α were more remarkable on migration rather than on proliferation (**Figure 11b**).

## Discussion

Several efforts are currently considered in order to design suitable materials for vascular regeneration. Natural polymers offer the advantage of reduced risk for tissue toxicity or inflammatory host immune responses [43]. Collagen, being the main natural ECM component, may be an ideal choice for an engineered scaffold material, as it has the advantage of providing both structural and microenviromental support. In the current study, we reported the use of collagen-gold nanocomposites with good biocompatibility and biological functions sutiable for vascular applications. In particular, these nanocomposites may provide as attractive nanobiomaterials for vascular tissue engineering. The larger fibers of 100 nm are more characteristic of hierarchically assembled type I collagen fibers in vitro and in vivo [41], [44]. The well interaction between collagen and AuNPs (43.5 ppm) resulted in surface roughness of ∼100 nm (larger fibers), which may be ascribed to the more organized and hierarchical assembly for collagen fibers in the presence of AuNPs. These biomimetic fibers may provide a microenvironment more favorable for adhesion, migration, and differentiation of MSCs.

When AuNPs are added in a polymer at the optimized concentration, a more favorable morphology is generated in the polymer [39], [45]. When the particle size of AuNPs is small, they are very effective in manipulating the interface of the composite, i.e. the amount of AuNPs required to change the microstructure may be low. In this situation, a higher concentration of AuNPs often leads to overloading and aggregation of nanoparticles, which adversely interferes with the microstructure and biological performances of the polymer [33], [34], [36]. In this study, the characteristic dimension of collagen in the presence of 43.5 ppm AuNPs was consistent with the favorable dimension in literature (∼100 nm), while the pure collagen revealed the less preferable dimension (∼15 nm). An excessive amount of AuNPs (174 ppm) reversed such an effect (∼32 nm). These results confirm that an optimized amount of AuNPs is critical for the functional improvement of collagen matrix. The morphology of pure Col was significantly altered by the addition at 43.5 ppm of AuNPs. We assumed that AuNPs may serve as the crosslinking point in the Col matrix. IR spectra indicated that there was interaction between Col and AuNPs. The decrease of the amide band intensity could be associated with the unwinding of the native collage triplehelical structure [40], [46].

For blood vessel tissue engineering, an ideal vascular scaffold should possess excellent biocompatibility and mechanical properties. AuNPs were demonstrated to have the ability to manipulate the microstructure of a synthetic or natural polymer even in very low concentration [33], [34], [36], [47]. The microstrucutural change of the polymer often led further to improved biological functions. Besides, the size of nanoparticles may be critical to their effects. For example, larger silica nanoparticles (80 nm) did not interact strongly with collagen, whereas smaller ones (12 nm) formed rosaries along the protein fibers [48]. Therefore, AuNPs (∼5 nm) were used in the current study and expected to interact with collagen. Vascular stents fabricated from the nanocomposites of collagen, poly(lactide-ε-caprolactone, and nano-hydroxyapatite (PCL/Col/nHA) by electrospinning had good mechanical properties, biocompatibility, and could guide tissue regeneration [49]. Nanocomposites may provide a new way to build scaffolds for vascularized-tissue engineering.

The morphogoical change of Col-Au 43.5 ppm increased the adhesion, proliferation, as well as migration of MSCs. Besides, Col-Au 43.5 ppm showed reduced inflammatory response and decreased platelet activation compared with the pure Col. These findings demonstrated that AuNPs had the potential to affect the biological performances of a matrix. Stem cell differentiation can be promoted by small molecules or appropriate ECM microenvironmental cues. In addition to providing a scaffolding support, ECM provides biological cues to the cell, activating cell signaling pathways that can trigger biological responses such as migration, proliferation, and survival [11]. However, neither the patterns of stem cell differentiation triggered by different ECM components nor the mechanisms mediating this differentiation are well known. MSCs are a particularly intriguing cell source for tissue engineering because of their capacity of differentiation and secretion of bioactive factors that are both immunomodulatory and trophic interest in their therapeutic potential [50]. In this study, we showed that Col-Au 43.5 ppm could modulate not only cellular morphological changes in MSCs, but also their functions. The mechanism by which nanotopography cues that affect stem cell behavior is thought to be related to the binding sites on ECMs and nanotopography-mediated cellular forces [51], [52]. We also observed that Col-Au 43.5 ppm could induce the the differentiation of MSCs into ECs. The promoted EC phenotype for MSCs cultured on Col-Au 43.5 ppm may make Col-Au nanocomposites suitable for vascular tissue regeneration applications.

Vascular tissue repair can be induced by a number of angiogenic cytokines, such as vascular endothelial growth factor (VEGF), stromal-derived factor-1α (SDF-1α) and basic fibroblast growth factor (bFGF) [53]. Among these factors, VEGF and SDF-1α are critical important in modulating or improving the process of vascular tissue repair. For example, interactions between SDF-1α and CXCR4 associate mobilization of MSCs into ischemic tissue [54]. The mobilization of MSCs subsequently VEGF and SDF-1α production by tissues can induce the formation of new blood vessels [55].

FAK is a middle molecule that regulates the cytoskeleton rearrangement, adhesion, and migration through activation of integrin. Previously we showed that addition of AuNPs into polyurethane, a non-biodegradble polmer, could trigger the αvβ3 integrin/FAK and PI3K/Akt/eNOS signaling pathways and modulate the migration of endothelial cells [33], [34]. In this work, the better stem cell functions on Col-Au nanocomposites were also associated with αvβ3/CXCR4 integrin/FAK/MMP-2 signaling pathway and regulation of Akt/eNOS protein expression. In particular, the αvβ3 integrin/CXCR4 expression levels for MSCs was enhanced by Col-Au 43.5 ppm, and further increased by VEG F (50 ng/ml) or SDF-1α (50 ng/ml). Since MSCs can secret VEGF to promote the recruitment of host MSCs through paracrine mechanism secret of SDF-1α from damage tissue [56], the ability of Col-Au nanocomposites to recruit MSCs may be an attractive feature of vascular tissue engineering biomaterials.

In summary, this study suggests that the properties of collagen biomaterials can be modulated by making composites with AuNPs. The property changes can reduce immune response and platelet activation. For stem cell cutlure, AuNPs in Col causes the cell behavioral changes through regulation of the αvβ3 integrin/CXCR4 receptor, FAK, MMP-2, and Akt/eNOS molecular signaling. The outcome is to promote the stem cell migration, proliferation, as well as differentiatin into endothelial cells. Our data are the first to report cellular interaction changes in response to Col-Au nanocomposites. Particularly, such understanding could improve the insight for engineering novel biological materials for vascular tissue applications.

## Conclusion

The biocompatibility of Col and Col-Au composites (containing 17.4, 43.5 and 174 ppm of AuNPs) and their effects on the behavior of MSCs were examined. Col-Au composites with 43.5 ppm of AuNPs could reduce the activation of monocytes and platelets as well as attenuate the ROS generation. The promoted adhesion, proliferation, and migration for MSCs grown on Col-Au 43.5 ppm may be associated with the modulation through VEGF/SDF-1α. This collagen nanocomposite may find applications in vasulcar tissue engineering.

## Supporting Information

Figure S1 **MSCs proliferation (by MTT assay) on control (TCPS), pure Col, and Col-Au nanocomposites containing 17.4** **ppm, 43.5** **ppm, and 174** **ppm of AuNPs after 24 and 72** **h of incubation.** *p<0.05, **p<0.01: greater than control (TCPS). The tendency was similar among different time points.
(TIF)
