# Supplementary figures and images for: In Vitro Study of a Novel Nanogold-Collagen Composite to Enhance the Mesenchymal Stem Cell Behavior for Vascular Regeneration
Source: PLoS One. 2014 Aug 5;9(8):e104019. doi: 10.1371/journal.pone.0104019 (PMC4122411; doi:10.1371/journal.pone.0104019)

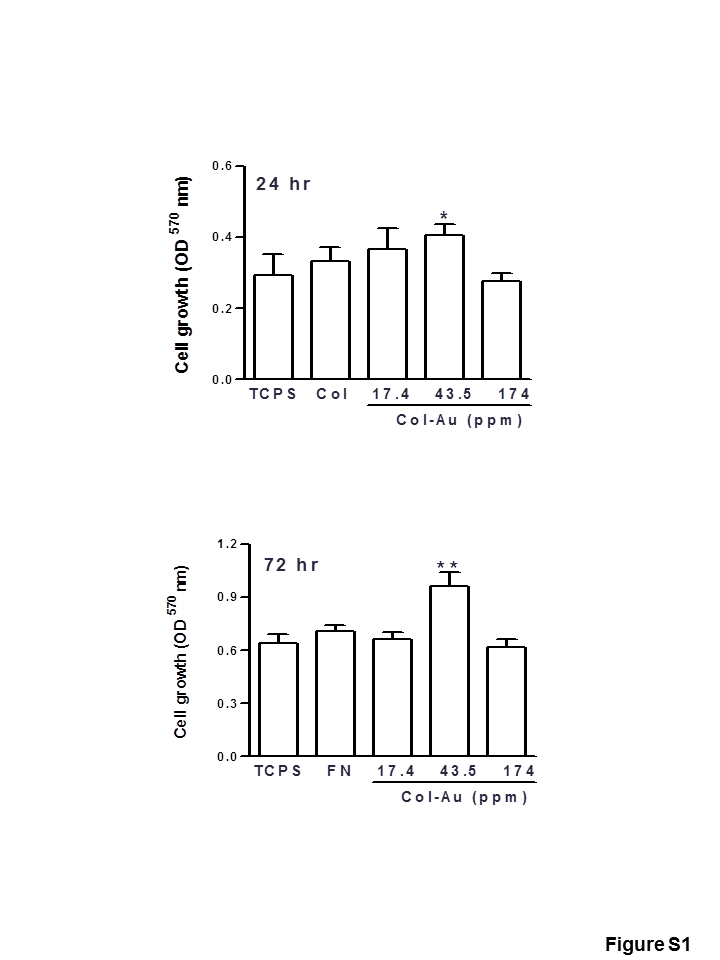

Supplement: Figure S1 — MSCs proliferation (by MTT assay) on control (TCPS), pure Col, and Col-Au nanocomposites containing 17.4 ppm, 43.5 ppm, and 174 ppm of AuNPs after 24 and 72 h of incubation. *p<0.05, **p<0.01: greater than control (TCPS). The tendency was similar among different time points. (TIF) [file pone.0104019.s001.tif]
